# Hematology and Serum Biochemistry Reference Intervals for Captive‐Born Owl Monkeys (*Aotus nancymae*): Effects of Age and Sex

**DOI:** 10.1111/jmp.70092

**Published:** 2026-06-26

**Authors:** Sarah M. Kezar, Sarah J. Neal, Gregory K. Wilkerson

**Affiliations:** ^1^ Department of Comparative Medicine, Michale E. Keeling Center for Comparative Medicine and Research The University of Texas MD Anderson Cancer Center Bastrop Texas USA; ^2^ Department of Pathology and Laboratory Medicine University of North Carolina Chapel Hill North Carolina USA

**Keywords:** American Society for Veterinary Clinical Pathology (ASVCP), *Aotus nancymai*, hematology, New World primate, nonhuman primate, serum chemistry

## Abstract

**Background:**

Owl monkeys (*Aotus* spp.) are a nocturnal nonhuman primate (NHP) native to central and South America that are used as infectious disease research models for human diseases, such as malaria and human immunodeficiency virus. Natural and infectious diseases may cause alterations in the hematology and serum biochemistry values, which necessitate the availability of reliable reference intervals for healthy animals.

**Methods:**

In this study, hematology and serum chemistry reference intervals for *Aotus nancymae* were calculated from 191 healthy animals (95 female, 96 male) and were generated based on age class (juvenile, adult, geriatric), sex (adults only), and across the entire sample.

**Results:**

Significant differences were observed in multiple parameters as a function of sex and age, some of which are inconsistent with existing data from *Aotus* spp. and other NHPs.

**Conclusions:**

The availability of age and sex specific reference intervals will be a valuable resource for monitoring the clinical health and effects of research interventions in owl monkeys.

## 1 Introduction

Owl monkeys are a nocturnal, New World, nonhuman primate (NHP) of the genus *Aotus* used primarily as an infectious disease model in biomedical research [1]. The most common owl monkey species bred and used for research in the United States is *Aotus nancymae (A. nancymae)*, which originates from the Amazonian region of Brazil, Colombia, and Peru [2]. While the spelling *Aotus nancymae* is used throughout this manuscript to remain consistent with other publications utilizing this colony, the species was originally named for Nancy Ma and spelled *Aotus nancymai* [3]. *Aotus nancymae* is an invaluable model for *Plasmodium* research and vaccine development, and has also been used for viral disease modeling, including Dengue, hepatitis, and HIV‐1 [1, 2, 4, 5, 6].

Despite the widespread use of owl monkeys in biomedical research, the hematology and biochemistry reference intervals commonly used and cited for *A*. *nancymae* were generated more than 30 years ago, using data primarily derived from wild‐caught animals of unconfirmed ages. Moreover, these reference intervals report only a subset of the hematology parameters and serum chemistry analytes commonly used in the field of laboratory animal medicine today, and most were produced using methods and instruments that lack the accuracy and reproducibility of those found in the modern day clinical pathology laboratory [1, 7, 8, 9]. Finally, animal care and husbandry of owl monkeys have evolved over time to include the increased usage of colony‐born animals, the elimination of many confounding health conditions, such as intestinal parasites, and an increased standardization of diet and housing conditions. As such, it is likely that reference intervals created over three decades ago may not be wholly representative of hematology and serum chemistry data acquired from contemporary owl monkey colonies. Updated reference intervals generated in accordance with professional society recommendations utilizing healthy, representative animals are needed for *A*. *nancymae* to provide adequate clinical care and accurate interpretation of research results.

This study describes the development of hematology parameter and serum chemistry analyte reference intervals in accordance with the American Society for Veterinary Clinical Pathology (ASVCP) guidelines for *A*. *nancymae* housed at the Keeling Center for Comparative Medicine and Research. Data were compiled by performing retrospective animal record reviews of hematology and serum chemistry data collected from 191 healthy, conscious, manually restrained animals aged 5 months to 30 years. Reference intervals were calculated for three age classes (juvenile, adult, and geriatric) as the middle 95% of values for each population of animals. Likewise, reference intervals were calculated for each sex across the entire sample, without regard to age demographic. Animals in the adult age demographic were additionally assessed for differences in their hematology parameters and serum chemistry values by sex.

## 2 Materials and Methods

### 2.1 Humane Care and Use Guidelines

All animals were housed at the University of Texas MD Anderson Cancer Center, Michale E. Keeling Center for Comparative Medicine and Research (KCCMR), in Bastrop, Texas, an AAALAC International accredited institution. All animals included in this study were covered by active protocols approved by the MD Anderson Institutional Animal Care and Use Committee (IACUC #0329‐RNXX).

### 2.2 Subjects

This study utilized data that had been previously collected as part of routine health assessments for *A*. *nancymae* housed within the owl monkey breeding colony at the KCCMR. This colony has been a closed colony since 2008. Between 2017 and 2023 when samples were collected for this study, the colony census ranged from 180 to 342 animals, and the extensive colony pedigree information was used to limit inbreeding. The 191 (95 females, 96 males) different animals selected for inclusion in the study were research naïve and appeared healthy, per their physical exam, bloodwork, and clinical history at, or near, the time point at which the blood samples were obtained. A single complete blood count (CBC) and serum chemistry analysis was included in the dataset for each animal. In cases where multiple datapoints were available for an animal, the earliest complete set of bloodwork for which the animal was clinically healthy was included in the dataset. Alopecia, mild dental disease, and mild osteoarthritis were common in aged animals and were not considered qualifications for exclusion from the study. Physical examinations included measurements of temperature, pulse, and respiration, and auscultation, palpation, and observation were performed by a specialized, clinical veterinarian. Animals demonstrating signs of clinical illness were excluded from the study population and scheduled for additional diagnostic testing. The 191 animals ranged in age from 5 months to 30 years (mean age = 6.6 years; standard deviation = 6.2 years) and were subcategorized by age into three groups: juveniles, adults, and geriatrics. Juveniles consisted of animals under 2 years of age (*n* = 47; 27 females, 20 males). The rationale for this age group was based on the earliest age of sexual maturity for KCCMR colony animals (~2 years old) and is consistent with the demographic cut‐off points previously used to describe age‐related differences in hematologic and biochemical parameters for other species of owl monkeys and marmosets [10, 11, 12]. Adults consisted of all study animals 2–12 years of age (*n* = 101; 49 females, 52 males) and represented the demographic with highest fecundity. Geriatrics consisted of animals greater than 12 years of age (*n* = 43; 19 females, 24 males). Rationale for the geriatrics age group was based on the observations that KCCMR animals tend to have decreased fecundity and increased age‐related clinical issues after 12 years of age. Our use of greater than 12 years of age for defining animals as geriatric is consistent with the cut‐off points used to describe post‐adult populations in some macaque papers investigating age‐related differences to hematologic and biochemical parameters [13, 14].

All animals were housed in holding rooms maintained at temperatures 78 ± 4 degrees F with a relative humidity of 30%–70% and a minimum of 10 complete air exchanges per hour. A 12:12 light dark cycle was maintained with an artificially shifted dawn and dusk to allow observation and husbandry activities for this nocturnal species during light/working hours. During the dark phase, dim red lighting was provided to allow for continued clinical observations. Animals were housed in socially compatible pairs or family groups unless separated due to compatibility issues. Housing spaces included 48″ D × 60.5″ H × 36″ W Britz cages and 28″ D × 31.5″ H × 32.75″ W stainless steel cages. Each cage had at least one thermoneutral, plastic nest‐box. Additional physical enrichment included ladders, chains, hanging manipulanda, and barrels. Caging was sanitized on a biweekly rotation. Animals were fed Teklad 7195, Hi‐Fiber Primate Diet. The base diet was supplemented with daily produce including oranges, sweet potato, peanuts, etc., and the regular fruit and vegetable rotation was supplemented with a forage mixture (low sugar cereal, nuts, seeds). Animals had ad libitum access to reverse osmosis treated water via an automated watering system. Animals were weighed and received 300 IU vitamin E via subcutaneous injection every 2 months for the prevention of vitamin‐E responsive anemia. Tape tests were performed annually on representative colony animals to confirm exclusion of pinworms from the colony. Bacterial and parasitic intestinal pathogen screening tests were routinely employed for any animal demonstrating clinical signs of intestinal disease.

### 2.3 Blood Collection and Analysis

Blood samples were collected from the femoral vein of conscious, manually restrained animals between June 2017 and October 2023 [15]. Blood was placed in K2EDTA‐treated and serum separator vacuum tubes (Becton Dickinson, Franklin Lakes, NJ) for hematology and serum chemistry analysis, respectively. Hematology and serum chemistry analyses were performed on the same day as the collections.

Hematology analysis was performed using a Siemens Advia 120 multi‐parameter automated hematology analyzer (Siemens, Tarrytown, NY) with the gating optimized for the *Aotus* species. Measured parameters included: red blood cell count (RBC, 10^6^/μL), hemoglobin (HGB, g/dL), hematocrit (HCT, %), mean corpuscular volume (MCV, fL), mean corpuscular hemoglobin (MCH, pg), mean corpuscular hemoglobin concentration (MCHC, g/dL), red blood cell distribution width (RDW, %), white blood cell count (WBC, 10^3^/μL), absolute neutrophil count (NEUT, 10^3^/μL), absolute lymphocyte count (LYMP, 10^3^/μL), absolute monocyte count (MONO, 10^3^/μL), absolute eosinophil count (EOS, 10^3^/μL), absolute basophil count (BASO, 10^3^/μL), relative neutrophil percentage (NEUT %), relative lymphocyte percentage (LYMP %), relative percentage monocyte (MONO %), relative eosinophil percentage (EOS %), relative basophil percentage (BASO %), mean platelet count (PLT, 10^3^/μL), platelet volume (MPV, fL).

Serum chemistry analysis was performed using a Beckman‐Coulter AU680 chemistry analyzer (Beckman Coulter, Brea, CA). Blood was allowed to clot at room temperature for 30–60 min before centrifugation at 1300 *g* for 10 min to obtain serum. Measured analytes included: alanine aminotransferase (ALT, IU/L), alkaline phosphatase (ALP, IU/L), aspartate aminotransferase (AST, IU/L), gamma‐glutamyltransferase (GGT, IU/L), total bilirubin (TBIL, mg/dL), lactate dehydrogenase (LDH, IU/L), creatine kinase (CK, IU/L), blood urea nitrogen (BUN, mg/dL), creatinine (CREA, mg/dL), calcium (CA, mg/dL), phosphorus (PHOS, mg/dL), glucose (GLUC, mg/dL), sodium (NA, mEq/L), potassium (K, mEq/L), chloride (CL, mEq/L), total carbon dioxide (TCO2, mEq/L), anion gap (AGAP, mEq/L), osmolality (OSMO, mOsm/kg), total protein (TP, g/dL), albumin (ALB, g/dL), globulins (GLOB, g/dL), albumin/globulin ratio (AGR, g/dL), cholesterol (CHOL, mg/dL), triglycerides (TRIG, mg/dL), iron (FE, μg/dL), total iron binding capacity (TIBC, μg/dL), unbound iron binding capacity (UIBC, μg/dL). Potassium, LDH, and AST values were excluded from the dataset for plasma samples noted to have appreciable hemolysis.

### 2.4 Statistical Analysis

Data processing methods were selected based on ASVCP guidelines for reference interval generation [16]. Statistical analysis was performed using Microsoft Excel (v 2402) and IBM SPSS Statistics for Windows version 24 (Armonk, NY). First, histograms of the data were plotted to assess the shape of the distribution of each parameter. Tukey's method with box plot construction was used to identify extreme outliers (Q1‐3IQR and Q3+3IQR) [17]. After removal of extreme outliers (Table 1), histogram and boxplot construction were repeated up to three times to remove additional extreme outliers when visual inspection of the histogram showed a natural break in the data and far outliers were consistent with clinical disease states [16]. Values that were statistically identified as extreme outliers but were considered physiologically relevant were not excluded from the dataset. A total of 72 of 8813 datapoints collected were found to be extreme outliers and were excluded from consideration (Table 1). These extreme outlier values are considered to be valid and accurate and not the result of measurement error. While it is possible these values may represent extreme variances from the average values in healthy animals, it is also plausible that these results could be an indicator of an underlying health condition to specific organ systems or could be the result of stress. Given that the goal of this study was to generate reference intervals that can be used to help assess animal health and identify underlying disease conditions, we opted to exclude the extreme outlier values from analyses although they are reported in Table 1 for reference.

**TABLE 1:** Hematology and serum chemistry descriptive statistics across the entire sample, including sample size (*N*), mean, standard deviation (SD), standard error of the mean (SEM), median, minimum value (min), maximum value (max), 95% reference interval (RI), and far outliers removed for each parameter.

	*N*	Mean	SD	SEM	Median	Min	Max	95% RI	Far outliers
RBC (10^6^/μL)	191	6.23	0.76	0.06	6.2	4.23	7.99	4.60–7.77	—
HGB (g/dL)	191	16.4	1.9	0.1	16.3	10.0	20.0	12.5–20.0	—
HCT (%)	191	51.4	6.0	0.4	51.3	34.0	65.0	40.0–62.1	—
MCV (fL)	191	82.8	4.5	0.3	82.1	70.0	98.0	74.8–92.4	—
MCH (pg)	191	26.4	1.6	0.1	26.4	23.0	32.0	23.6–29.5	—
MCHC (g/dL %)	191	32.0	1.3	0.1	31.9	29.0	35.0	29.3–34.8	—
RDW (%)	190	13.6	1.0	0.1	13.4	11.2	17.1	11.9–15.9	19.1
WBC (10^3^/μL)	191	8.02	2.88	0.21	7.67	2.06	17.18	3.30–15.32	—
NEUT (10^3^/μL)	185	2.0	1.1	0.1	1.7	0.2	5.5	0.5–5.0	9.19; 7.45; 6.44; 6.21
LYMP (10^3^/μL)	189	5.6	2.4	0.2	5.2	2.0	15.0	2.1–11.8	—
MONO (10^3^/μL)	178	0.09	0.05	0.00	0.08	0.01	0.26	0.02–0.22	0.6; 0.54; 0.43; 0.35: 0.34; 0.33; 0.32; 0.32; 0.32; 0.31; 0.28
EOS (10^3^/μL)	186	0.18	0.14	0.01	0.155	0	0.65	0.01–0.56	2.33; 0.95; 0.8
BASO (10^3^/μL)	184	0.05	0.03	0.00	0.04	0.01	0.18	0.01–0.14	0.52; 0.26; 0.25; 0.25; 0.22; 0.21
NEUT (%)	185	25.4	11.3	0.8	23.4	5.9	53.7	8.3–51.8	—
LYMP (%)	189	69.3	13.5	1.0	72.3	28.0	92.0	36.5–89.2	—
MONO (%)	173	1.1	0.6	0.0	1.0	0.1	3.2	0.2–2.7	4.85; 4.74; 4.71; 3.58; 3.35
EOS (%)	185	2.3	1.6	0.1	1.9	0.0	8.5	0.2–6.2	9.82
BASO (%)	182	0.6	0.3	0.0	0.5	0.2	1.8	0.2–1.6	2.94; 2.14
PLT (10^3^/μL)	188	375	125	9	374	88	864	119–623	—
MPV (fL)	188	10.8	1.7	0.1	10.7	7.9	16.4	8.4–14.8	—
ALT (IU/L)	190	73	25	2	69	23	154	33–133	229
ALP (IU/L)	184	410	344	25	297	39	1570	58–1341	2576; 2061; 2032; 2031; 1951; 1635; 1618
AST (IU/L)	182	163	60	4	149	59	398	86–343	—
GGT (IU/L)	181	14	12	1	10	3	65	4–59	576; 379; 372; 262; 179; 139; 121; 85; 82
TBIL (mg/dL)	190	0.32	0.09	0.01	0.31	0.12	0.60	0.15–0.50	0.9
LDH (IU/L)	182	289	115	9	266	111	710	129–594	804
CK (IU/L)	181	638	649	48	366	56	2891	67–2567	9791; 7186; 6173; 5234; 4859; 4722; 3892; 3716; 3583; 3209
BUN (mg/dL)	191	16	6	0	16	4	34	6–31	—
CREA (mg/dL)	184	0.55	0.13	0.01	0.53	0.30	1.00	0.30–0.84	—
Ca (mg/dL)	191	10.0	0.8	0.1	10.0	8.0	12.9	8.4–11.7	—
PHOS (mg/dL)	189	4.7	1.5	0.1	4.5	1.6	10.5	2.1–8.5	13
GLUC (mg/dL)	191	120	38	3	112	48	246	59–212	—
Na (meq/L)	191	147	4	0	147	135	158	139–155	—
K (meq/L)	184	4.1	0.7	0.0	4.1	2.4	6.7	2.9–5.4	—
Cl (meq/L)	191	102	4	0	103	92	114	93–111	—
TCO2 (mEq/L)	191	16.7	5.7	0.4	18.0	2.0	32.0	5.0–27.2	—
AGAP (mEq/L)	191	31.8	7.6	0.5	31.0	16.0	56.0	20.0–50.2	—
Osmo (mOsm/kg)	191	285.9	7.8	0.6	285.0	262.0	310.1	270.8–304.0	—
TP (g/dL)	189	7.5	0.8	0.1	7.5	5.1	9.8	5.6–9.2	—
ALB (g/dL)	191	4.3	0.6	0.0	4.3	2.5	5.7	2.8–5.3	—
GLOB (mg/dL)	187	3.1	0.5	0.0	3.1	2.0	4.7	2.3–4.5	6.8; 6.0
AGR (g/dL)	189	1.4	0.3	0.0	1.4	0.4	2.0	0.8–1.9	—
CHOL (mg/dL)	190	110	31	2	109	44	232	59–173	251
TRIG (mg/dL)	184	155	60	4	149	29	363	63–324	930; 822; 566; 478; 399
FE (μg/dL)	189	127	42	3	121	46	294	61–222	357
TIBC (μg/dL)	190	386	66	5	383	233	592	252–547	—
UIBC (μg/dL)	190	258	65	5	259	110	437	124–394	—

Following outlier removal, descriptive statistics (i.e., mean, standard deviation, standard error of the mean, median, minimum, and maximum values) were calculated for (i) the entire dataset, (ii) separately for both sexes (females and males), and (iii) separately across age demographics (juvenile, adult, geriatric). The same descriptive statistics were also calculated for male and female animals within the adult demographic, although this analysis was not possible for the juvenile or geriatric animals due to the smaller size of these two demographics. Most data were normally distributed, but some parameters were skewed, and subgroup sample sizes were unequal. Therefore, we opted to use nonparametric statistical methods with bootstrapping when calculating reference intervals to maintain a consistent statistical approach across all parameters [16]. In accordance with ASVCP recommendations, the nonparametric method was used to determine reference intervals with the lower limit corresponding to the 2.5th fractile and the upper limit corresponding to the 97.5th fractile for all parameters with a sample size of 40 or greater. Reference intervals for groups with a sample size less than 40 were defined as the minimum and maximum of the available dataset. We also used bootstrapped *t*‐tests and one‐way ANOVAs to explore differences between sexes and age classes, respectively [18]. We confirmed that age was not a confound in the analyses examining differences as a function of sex using a *t*‐test, which showed that males and females did not differ significantly in age (*p* > 0.05).

## 3 Results

### 3.1 Descriptive Statistics and Reference Ranges

Table 1 shows the final sample size, mean, standard deviation, standard error, median, maximum, minimum, 95% reference interval, and far outliers for each of the hematology and serum chemistry parameters for the overall population. Sex‐ and age‐class specific reference intervals for blood analytes were also calculated (Tables 2, 3, 4, 5, 6, 7).

**TABLE 2:** Hematology descriptive statistics as a function of sex across the entire sample, including sample size (*N*), mean, median, 95% reference interval (RI), and results of independent samples *t*‐tests between males and females.

	Female RI					Male RI					*t*	df	*p*
	*N*	Mean	SD	Median	95% RI	*N*	Mean	SD	Median	95% RI			
RBC (10^6^/μL)	95	6.11	0.77	6.11	4.67–7.78	96	6.35	0.74	6.34	4.53–7.77	−2.2	189	0.03*
HGB (g/dL)	95	16.1	2.0	15.9	12.3–19.9	96	16.7	1.9	17.1	12.4–20.1	−2.36	189	0.02*
HCT (%)	95	50.4	6.1	50.2	39.9–63.1	96	52.5	5.7	52.8	40.5–62.2	−2.44	189	0.02*
MCV (fL)	95	82.7	4.5	82.0	74.9–92.5	96	82.9	4.4	82.3	73.6–93.4	−0.26	189	0.80
MCH (pg)	95	26.4	1.4	26.2	23.8–29.3	96	26.5	1.7	26.6	23.3–29.8	−0.17	189	0.87
MCHC (g/dL %)	95	32.0	1.3	31.9	29.3–34.9	96	31.9	1.3	31.8	29.2–34.8	0.19	189	0.85
RDW (%)	94	13.6	1.2	13.4	11.6–16.8	96	13.5	0.8	13.4	12.1–15.5	0.37	163.04	0.71
WBC (10^3^/μL)	95	8.07	2.99	8.2	3.19–15.11	96	7.96	2.78	7.49	3.52–5.68	0.27	189	0.78
NEUT (10^3^/μL)	93	2.0	1.0	1.7	0.5–4.7	92	2.0	1.2	1.7	0.4–5.3	−0.01	183	1.00
LYMP (10^3^/μL)	95	5.6	2.4	5.8	1.2–11.2	94	5.5	2.5	5.0	2.4–13.3	−0.21	187	0.83
MONO (10^3^/μL)	91	0.09	0.05	0.08	0.01–0.23	87	0.09	0.04	0.09	0.02–0.21	0.1	170.85	0.92
EOS (10^3^/μL)	93	0.18	0.14	0.16	0.01–0.58	93	0.18	0.14	0.15	0.01–0.54	−0.15	184	0.88
BASO (10^3^/μL)	93	0.05	0.03	0.05	0.01–0.15	91	0.05	0.03	0.04	0.01–0.16	0.11	182	0.91
NEUT (%)	93	25.5	10.6	25.2	8.8–49.2	92	25.3	11.9	22.6	8.1–52.5	0.16	183	0.87
LYMP (%)	95	69.1	13.1	70.3	37.7–88.1	94	69.5	13.9	73.4	35.8–89.6	0.17	187	0.87
MONO (%)	86	1.0	0.6	0.9	0.2–2.8	87	1.2	0.5	1.1	0.2–2.7	−1.86	171	0.06
EOS (%)	93	2.3	1.7	2.0	0.1–5.9	92	2.3	1.6	1.9	0.2–6.6	0.13	183	0.90
BASO (%)	92	0.6	0.3	0.5	0.2–1.6	90	0.6	0.3	0.5	0.2–1.6	0.26	180	0.79
PLT (10^3^/μL)	92	399	133	406	134–659	96	353	113	348	113–610	2.58	186	0.01*
MPV (fL)	92	10.7	1.7	10.5	8.4–14.6	96	10.9	1.6	10.8	8.2–15.5	−1.07	186	0.28

* Value is significantly different between females and males (*p* < 0.05).

### 3.2 Effect of Sex

**FIGURE 1:**
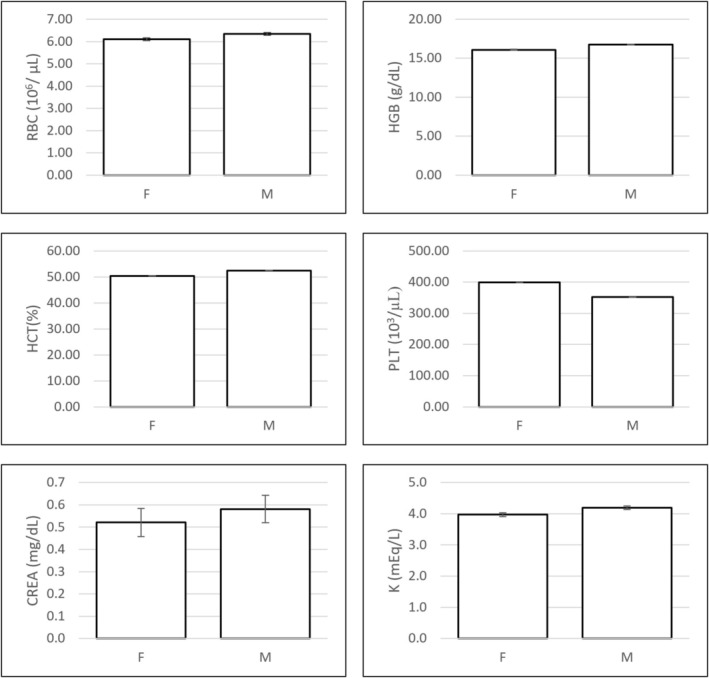
Hematology and serum chemistry analytes that differed between males and females of the entire population.

The mean values of four hematologic parameters and two serum chemistry analytes differed significantly between male and female owl monkeys when considering the entire study population: RBC, HGB, HCT, PLT, CREA, and K. The mean values of RBC, HGB, HCT, CREA, and K were significantly higher in males than females, while PLT mean values were higher in females compared to males (Tables 2 and 3, Figure 1).

**TABLE 3:** Serum chemistry descriptive statistics as a function of sex across the entire sample, including sample size (*N*), mean, median, 95% reference interval (RI), and results of independent samples *t*‐tests between males and females.

	Female RI					Male RI					*t*	df	*p*
	*N*	Mean	SD	Median	95% RI	*N*	Mean	SD	Median	95% RI			
ALT (IU/L)	94	70	25	67	26–134	96	75	25	70	34–139	−1.26	188	0.21
ALP (IU/L)	91	426	354	314	62–1412	93	393	335	287	51–1350	0.65	182	0.51
AST (IU/L)	90	163	61	147	86–358	92	164	59	152	84–322	−0.07	180	0.94
GGT (IU/L)	89	13	13	9	3–62	92	15	11	11	5–50	−1.11	179	0.27
TBIL (mg/dL)	94	0.31	0.10	0.30	0.13–0.51	96	0.32	0.08	0.32	0.19–0.54	−0.97	179.76	0.33
LDH (IU/L)	90	296	113	275	135–594	92	283	118	253	126–632	0.8	180	0.43
CK (IU/L)	90	675	661	404	71–2721	91	601	639	345	62–2548	0.76	179	0.45
BUN (mg/dL)	95	15	7	15	5–32	96	16	5	16	7–31	−0.94	179.48	0.35
CREA (mg/dL)	92	0.52	0.11	0.5	0.32–0.78	92	0.58	0.14	0.58	0.29–0.86	−3.25	182	0.001*
Ca (mg/dL)	95	10.0	0.9	10.0	8.3–11.8	96	10.1	0.7	10.1	8.5–11.6	−0.38	189	0.71
PHOS (mg/dL)	94	4.7	1.7	4.4	1.8–9.0	95	4.8	1.4	4.5	2.2–8.2	−0.37	187	0.71
GLUC (mg/dL)	95	122	37	113	60–213	96	117	39	110	55–220	0.94	189	0.35
Na (meq/L)	95	147	3	147	140–155	96	146	4	146	138–155	1.54	189	0.13
K (meq/L)	91	4.0	0.7	3.9	2.8–5.4	93	4.2	0.6	4.3	2.9–5.4	−2.25	182	0.03*
Cl (meq/L)	95	102	5	103	93–112	96	103	4	103	94–111	−1.27	180.82	0.21
TCO2 (mEq/L)	95	16.9	5.9	18.0	4.4–28.6	96	16.5	5.5	17.0	6.0–27.0	0.5	189	0.62
AGAP (mEq/L)	95	32.3	7.6	31.0	21.0–52.6	96	31.3	7.5	31.0	19.0–49.2	0.95	189	0.35
osmo (mOsm/kg)	95	286.5	7.4	286.0	274.0–307.4	96	285.3	8.1	285.0	268.3–303.4	1.06	189	0.29
TP (g/dL)	94	7.5	0.8	7.5	5.6–9.0	95	7.4	0.8	5.1	5.7–9.5	0.53	187	0.60
ALB (g/dL)	95	4.3	0.6	4.3	2.8–5.4	96	4.3	0.6	4.3	2.9–5.3	0.68	189	0.50
GLOB (mg/dL)	93	3.2	0.5	3.1	2.3–4.4	94	3.1	0.5	3.1	2.3–4.6	0.4	185	0.69
AGR (g/dL)	94	1.4	0.3	1.4	0.7–2.0	95	1.4	0.3	1.4	0.7–1.8	0.1	187	0.92
CHOL (mg/dL)	94	108	30	108	57–163	96	113	31	111	60–189	−1.19	188	0.24
TRIG (mg/dL)	92	156	62	148	63–333	92	153	57	151	63–310	0.31	182	0.76
FE (μg/dL)	94	124	35	120	64–201	95	131	48	124	56–262	−1.08	172.09	0.28
TIBC (μg/dL)	94	387	70	383	235–569	96	385	61	381	272–546	0.24	188	0.81
UIBC (μg/dL)	94	263	65	261	131–417	96	252	65	258	115–386	1.19	188	0.23

* Value is significantly different between females and males (*p* < 0.05).

**FIGURE 2:**
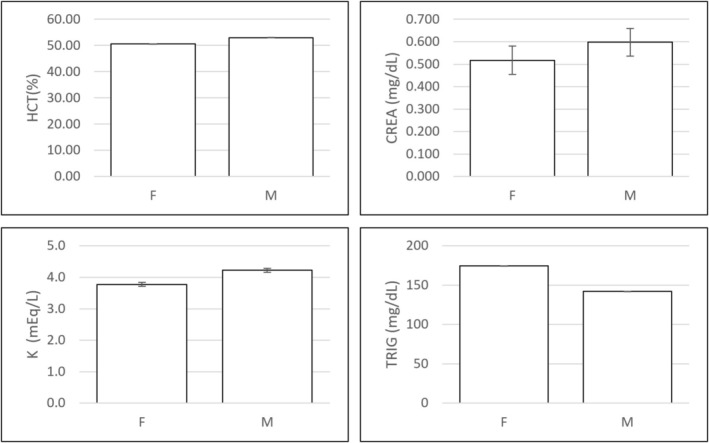
Hematology and serum chemistry analytes that differed between adult male and female owl monkeys.

When sex was evaluated in the adult demographic alone, the mean values of one hematologic parameter and three serum chemistry analytes differed significantly between male and female owl monkeys: HCT, CREA, K, and TRIG. The mean values HCT, CREA, and K were higher in males than females, and TRIG mean values were higher in females than males (Tables 4 and 5, Figure 2).

**TABLE 4:** Hematology descriptive statistics as a function of sex only in the adult age class, including sample size (*N*), mean, median, 95% reference interval (RI), and results of independent samples *t*‐tests between juveniles (Juv), adults (Ad), and geriatrics (Ger).

	Female RI					Male RI					*t*	df	*p*
	*N*	Mean	SD	Median	95% RI	*N*	Mean	SD	Median	95% RI			
RBC (10^6^/μL)	49	6.13	0.85	6.17	4.32–7.82	52	6.40	0.72	6.37	4.45–7.78	−1.748	99	0.084
HGB (g/dL)	49	16.2	2.2	16.0	12.1–19.9	52	17.0	1.9	17.3	10.8–20.1	−1.842	99	0.068
HCT (%)	49	50.6	6.6	50.2	39.5–63.7	52	53.0	5.5	53.5	35.9 62.3	−2.013	99	0.047*
MCV (fL)	49	82.8	4.9	82.8	73.5–93.9	52	83.0	4.4	82.7	72.1–93.6	−0.231	99	0.818
MCH (pg)	49	26.6	1.5	26.5	24.0–29.3	52	26.6	1.7	26.6	23.3–29.8	−0.112	99	0.911
MCHC (g/dL %)	49	32.1	1.2	32.1	29.8–35.2	52	32.0	1.3	32.2	29.1–35.0	0.247	99	0.805
RDW (%)	48	13.8	1.4	13.6	11.3–17.0	52	13.6	0.8	13.7	12.2–15.7	0.953	98	0.343
WBC (10^3^/μL)	49	8.26	3.30	8.76	2.64–16.40	52	7.76	3.12	7.45	2.40–16.79	0.786	99	0.434
NEUT (10^3^/μL)	49	2.1	1.1	2.0	0.3–4.8	52	1.9	1.1	1.6	0.3–5.2	1.025	99	0.308
LYMP (10^3^/μL)	49	5.8	2.8	5.8	1.7–13.4	52	5.6	2.8	4.9	2.0–14.8	0.418	99	0.677
MONO (10^3^/μL)	47	0.09	0.06	0.09	0.01–0.25	50	0.09	0.04	0.09	0.02–0.21	0.263	84.60	0.793
EOS (10^3^/μL)	48	0.18	0.14	0.15	0.00–0.59	52	0.16	0.12	0.13	0.00–0.52	0.76	98	0.449
BASO (10^3^/μL)	48	0.06	0.04	0.05	0.01–0.15	48	0.05	0.04	0.04	0.01–0.18	0.693	94	0.490
NEUT %	49	25.3	11.3	25.6	7.3–51.2	52	24.4	11.3	22.3	8.1–52.4	0.807	99	0.422
LYMP %	49	69.0	12.7	69.1	40.3–90.8	52	71.2	12.0	73.9	44.3–89.6	−0.873	99	0.385
MONO %	44	1.1	0.7	0.9	0.2–3.1	50	1.3	0.6	1.1	0.2–2.8	−1.74	92	0.085
EOS %	48	2.3	1.7	2.0	0.0–5.8	51	2.1	1.5	1.6	0.1–6.7	0.693	97	0.490
BASO %	47	0.7	0.4	0.6	0.3–1.8	47	0.7	0.4	0.5	0.2–1.7	0.399	92	0.691
PLT (10^3^/μL)	46	388	127	394	95–662	52	348	102	340	118–582	1.734	96	0.086
MPV (fL)	46	11.0	1.8	10.9	8.5–14.5	52	11.2	1.8	11.0	8.3–16.2	−0.658	96	0.512

* Value is significantly different between females and males (*p* < 0.05).

**TABLE 5:** Serum chemistry descriptive statistics as a function of sex only in the adult age class, including sample size (*N*), mean, median, 95% reference interval (RI), and results of independent samples *t*‐tests between juveniles (Juv), adults (Ad), and geriatrics (Ger).

	Female RI					Male RI					*t*	df	*p*
	*N*	Mean	SD	Median	95% RI	*N*	Mean	SD	Median	95% RI			
ALT (IU/L)	49	71	26	69	27–142	52	72	20	69	33–106	−0.25	99	0.803
ALP (IU/L)	49	289	155	289	56–634	52	317	208	291	23–1154	−0.75	99	0.455
AST (IU/L)	46	166	57	148	80–356	50	163	52	151	87–279	0.342	94	0.733
GGT (IU/L)	47	14	13	10	3–64	49	14	10	12	5–51	−0.029	94	0.977
TBIL (mg/dL)	48	0.32	0.10	0.31	0.12–0.50	52	0.34	0.08	0.33	0.2–0.59	−1.089	98	0.279
LDH (IU/L)	46	313	125	286	118–653	51	279	104	258	118–534	1.47	95	0.145
CK (IU/L)	46	585	516	366	64–1871	50	498	462	277	72–1760	0.874	94	0.384
BUN (mg/dL)	49	13	5	13	5–25	52	15	5	15	6–29	−1.43	99	0.156
CREA (mg/dL)	46	0.52	0.10	0.49	0.33–0.76	48	0.60	0.13	0.59	0.35–0.97	−3.304	92	0.001*
Ca (mg/dL)	49	9.9	1.0	9.9	8.3–12.6	52	10.0	0.8	10.2	8.4–11.8	−0.618	99	0.538
PHOS (mg/dL)	48	4.6	1.5	4.5	1.8–8.9	52	4.7	1.5	4.3	2.3–9.7	−0.077	98	0.939
GLUC (mg/dL)	49	131	43	125	51–214	52	119	45	114	49–243	1.366	99	0.175
Na (meq/L)	49	147	3	147	139–154	52	147	4	148	139–157	−0.143	99	0.886
K (meq/L)	47	3.8	0.7	3.6	2.5–5.3	51	4.2	0.6	4.3	2.9–5.2	−3.564	96	0.001*
Cl (meq/L)	49	102	5	103	93–109	52	103	3	102	94–112	−1.226	89.389	0.224
TCO2 (mEq/L)	49	15.9	2.3	17.0	4.0–27.8	52	15.8	5.9	17.0	3.3–26.4	0.092	99	0.927
AGAP (mEq/L)	49	33.3	8.5	32.0	20.5–55.3	52	33.0	7.9	33.0	19.0–50.0	0.21	99	0.834
Osmo (mOsm/kg)	49	287.0	8.5	287.0	268.8–310.0	52	287.3	8.0	287.0	272.2–304.3	−0.186	99	0.853
TP (g/dL)	48	7.6	0.8	7.6	5.5–9.2	51	7.6	0.8	7.5	5.7–9.5	0.41	97	0.968
ALB (g/dL)	49	4.3	0.7	4.3	2.7–5.6	52	4.3	0.6	4.4	2.7–5.5	−0.196	99	0.845
GLOB (mg/dL)	47	3.2	0.4	3.2	2.5–4.2	51	3.2	0.5	3.2	2.4–4.7	−0.024	96	0.981
AGR (g/dL)	48	1.3	0.3	1.3	0.5–1.9	51	1.4	0.3	1.4	0.7–1.8	−0.642	97	0.523
CHOL (mg/dL)	49	109	33	108	48–215	52	111	26	109	61–166	−0.385	99	0.701
TRIG (mg/dL)	48	175	69	162	38–357	51	142	41	139	67–242	2.82	75.73	0.006*
FE (μg/dL)	48	125	35	122	65–210	52	131	46	124	49–264	−0.691	98	0.491
TIBC (μg/dL)	48	380	71	378	241–567	52	378	63	370	248–548	0.125	98	0.9
UIBC (μg/dL)	48	255	65	252	142–431	52	247	66	250	113–407	0.563	98	0.575

* Value is significantly different between females and males (*p* < 0.05).

### 3.3 Effect of Age

**FIGURE 3:**
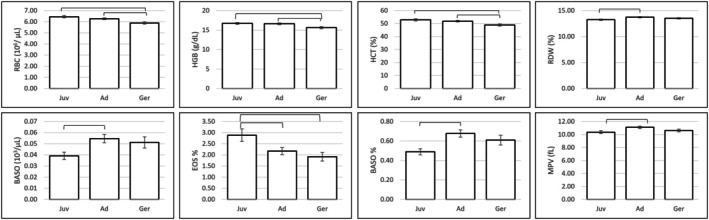
Hematology analytes that showed significant differences across age groups.

**FIGURE 4:**
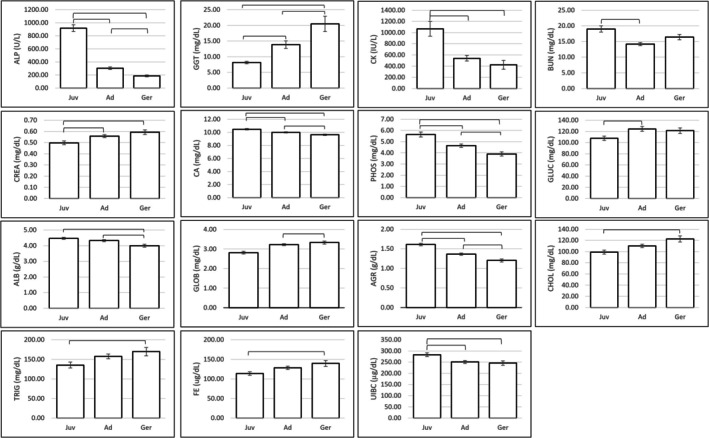
Serum chemistry analytes that showed significant differences across age groups.

The mean values of eight hematology parameters differed significantly between two or more age classes: RBC, HGB, HCT, RDW, BASO, EOS %, BASO %, and MPV (Table 6, Figure 3). The mean values of 15 serum chemistry analytes differed significantly between two or more age classes: ALP, GGT, CK, BUN, CREA, CA, PHOS, GLUC, ALB, GLOB, AGR, CHOL, TRIG, FE, and UIBC (Table 7, Figure 4).

**TABLE 6:** Hematology descriptive statistics as a function of age class across the entire sample, including sample size (N), mean, median, 95% reference interval (RI), and results of independent samples *t*‐tests between juveniles (Juv), adults (Ad), and geriatrics (Ger).

		*N*	Mean	SD	Median	95% RI	*F*	df	*p*
RBC (10^6^/μL)	Juv	47	6.45	0.68	6.47	5.31–7.95	6.753	2, 188	0.001^b,c^
	Ad	101	6.27	0.80	6.33	4.50–7.80			
	Ger	43	5.89	0.66	5.94	4.50–7.43			
HGB (g/dL)	Juv	47	16.7	1.6	16.3	14.1–20.3	4.947	2, 188	0.008^b,c^
	Ad	101	16.6	2.1	16.8	11.9–20.0			
	Ger	43	15.6	1.8	15.5	12.8–18.9			
HCT (%)	Juv	47	52.9	5.3	52.1	41.5–64.1	5.553	2, 188	0.005^b,c^
	Ad	101	51.8	6.2	51.8	39.7–62.2			
	Ger	43	48.9	5.6	47.8	41.1–62.7			
MCV (fL)	Juv	47	82.1	3.8	81.9	73.5–90.7	0.779	2, 188	0.46
	Ad	101	82.9	4.6	82.8	74.2–93.4			
	Ger	43	83.3	4.8	82.4	74.3–97.6			
MCH (pg)	Juv	47	26.0	1.3	26.0	23.4–29.3	2.3	2, 188	0.103
	Ad	101	26.6	1.6	26.5	23.5–29.6			
	Ger	43	26.6	1.7	26.4	23.2–31.5			
MCHC (g/dL %)	Juv	47	31.7	1.3	31.7	28.9–34.9	1.363	2, 188	0.258
	Ad	101	32.1	1.2	32.1	29.6–34.9			
	Ger	43	31.9	1.4	31.9	29.2–34.8			
RDW (%)	Juv	47	13.2	0.7	13.2	11.6–15.1	3.998	2, 187	0.02^a^
	Ad	100	13.7	1.1	13.6	11.8–16.7			
	Ger	43	13.5	0.8	13.4	12.2–15.8			
WBC (10^3^/μL)	Juv	47	7.80	2.47	7.67	3.25–13.33	0.335	2, 188	0.716
	Ad	101	8.00	3.20	7.58	2.79–16.53			
	Ger	43	8.29	2.53	7.96	3.64–15.41			
NEUT (10^3^/μL)	Juv	45	1.9	1.1	1.6	0.4–5.3	0.275	2, 182	0.76
	Ad	101	2.0	1.1	1.7	0.4–4.9			
	Ger	39	2.1	1.2	1.7	0.6–5.5*			
LYMP (10^3^/μL)	Juv	47	5.2	2.0	4.9	2.0–11.1	0.56	2, 186	0.572
	Ad	101	5.7	2.8	5.0	1.8–13.9			
	Ger	41	5.7	1.9	5.4	2.1–10.2			
MONO (10^3^/μL)	Juv	42	0.09	0.05	0.08	0.01–0.22	0.727	2, 175	0.485
	Ad	97	0.09	0.05	0.09	0.01–0.22			
	Ger	39	0.08	0.04	0.07	0.03–0.25*			
EOS (10^3^/μL)	Juv	45	0.22	0.16	0.19	0.02–0.64	2.111	2, 183	0.124
	Ad	100	0.17	0.13	0.14	0.01–0.54			
	Ger	41	0.17	0.13	0.14	0.01–0.53			
BASO (10^3^/μL)	Juv	47	0.04	0.02	0.04	0.01–0.11	3.598	2, 181	0.029^a^
	Ad	96	0.05	0.04	0.04	0.01–0.16			
	Ger	41	0.05	0.03	0.04	0.01–0.15			
NEUT %	Juv	45	25.3	11.4	24.4	6.6–51.4	2.334	2, 182	0.982
	Ad	101	25.3	11.3	23.4	8.2–50.6			
	Ger	39	25.7	11.3	23.3	8.3–52.8*			
LYMP %	Juv	47	67.9	15.2	71.1	29.5–89.8	0.478	2, 186	0.621
	Ad	101	70.1	12.3	71.0	42.9–89.5			
	Ger	41	68.9	14.2	73.5	31.1–89.1			
MONO %	Juv	40	1.1	0.5	1.0	0.1–2.6	1.495	2, 170	0.227
	Ad	94	1.2	0.6	1.0	0.2–2.9			
	Ger	39	1.0	0.4	1.0	0.4–2.3*			
EOS %	Juv	45	2.9	1.9	2.5	0.2–8.4	4.49	2, 182	0.012^a,b^
	Ad	99	2.2	1.6	1.7	0.0–6.0			
	Ger	41	1.9	1.3	1.8	0.2–5.7			
BASO %	Juv	47	0.5	0.2	0.5	0.2–0.9	5.385	2, 179	0.005^a^
	Ad	94	0.7	0.4	0.5	0.2–1.7			
	Ger	41	0.6	0.3	0.5	0.2–1.5			
PLT (10^3^/μL)	Juv	47	401	129	400	117–810	1.342	2, 185	0.264
	Ad	98	367	116	351	118–624			
	Ger	43	367	141	366	108–657			
MPV (fL)	Juv	47	10.4	1.7	10.1	8.0–15.8	3.739	2, 185	0.026^a^
	Ad	98	11.1	1.8	10.9	8.4–15.5			
	Ger	43	10.6	1.3	10.6	8.1–13.6			

*Note:* Value is significantly different between groups (*p* < 0.05; a; juvenile and adult, b; juvenile and geriatric, c; adult and geriatric).

* Minimum and Maximum from dataset are used in lieu of calculated RI minimum and maximum.

**TABLE 7:** Serum chemistry descriptive statistics as a function of age class across the entire sample, including sample size (*N*), mean, median, 95% reference interval (RI), and results of independent samples *t*‐tests between juveniles (Juv), adults (Ad), and geriatrics (Ger).

		*N*	Mean	SD	Median	95% RI	*F*	df	*p*
ALT (IU/L)	Juv	46	77	21	76	40–129	0.88	2, 187	0.418
	Ad	101	72	23	69	32–128			
	Ger	43	71	32	62	23–153			
ALP (IU/L)	Juv	40	919	330	899	345–1567	155.01	2, 181	< 0.001^a,b,c^
	Ad	101	303	184	289	53–677			
	Ger	43	185	92	157	52–443			
AST (IU/L)	Juv	44	168	60	158	86–361	0.40	2, 179	0.668
	Ad	96	164	54	149	85–282			
	Ger	42	157	71	134	61–394			
GGT (IU/L)	Juv	46	8	3	8	3–14	12.83	2, 178	< 0.001^a,b,c^
	Ad	96	14	12	11	4–57			
	Ger	39	21	15	16	4–61*			
TBIL (mg/dL)	Juv	47	0.31	0.09	0.29	0.14–0.58	1.91	2, 187	0.151
	Ad	100	0.33	0.09	0.33	0.15–0.54			
	Ger	43	0.30	0.09	0.29	0.13–0.52			
LDH (iu/L)	Juv	44	288	86	274	167–503	0.35	2, 179	0.705
	Ad	97	295	115	280	120–594			
	Ger	41	277	141	229	129–708			
CK (IU/L)	Juv	43	1066	864	785	120–2890	14.66	2, 178	< 0.001^a,b^
	Ad	96	540	488	337	66–1795			
	Ger	42	424	522	209	57–2558			
BUN (mg/dL)	Juv	47	19	7	17	7–34	11.53	2, 188	< 0.001^a^
	Ad	101	14	5	14	6–26			
	Ger	43	16	6	16	4–31			
CREA (mg/dL)	Juv	47	0.50	0.12	0.50	0.27–0.83	7.06	2, 181	0.001^a,b^
	Ad	94	0.56	0.12	0.53	0.35–0.86			
	Ger	43	0.59	0.14	0.61	0.29–0.84			
Ca (mg/dL)	Juv	47	10.5	0.6	10.5	8.3–11.8	13.54	2, 188	< 0.001^a,b,c^
	Ad	101	10.0	0.9	10.0	8.4–11.8			
	Ger	43	9.6	0.7	9.5	8.5–11.5			
PHOS (mg/dL)	Juv	46	5.6	1.5	5.9	2.6–9.5	16.84	2, 186	< 0.001^a,b,c^
	Ad	100	4.6	1.5	4.4	2.1–8.7			
	Ger	43	3.9	1.2	3.9	1.6–8.4			
GLUC (mg/dL)	Juv	47	108	26	103	63–190	3.21	2, 188	0.043^a^
	Ad	101	124	44	123	51–225			
	Ger	43	121	33	118	62–209			
Na (meq/L)	Juv	47	146	4	146	138–155	2.43	2, 188	0.091
	Ad	101	147	4	148	140–154			
	Ger	43	146	4	146	135–155			
K (meq/L)	Juv	44	4.3	0.7	4.3	2.9–6.6	2.69	2, 181	0.071
	Ad	98	4.0	0.7	4.0	2.7–5.2			
	Ger	42	4.1	0.7	4.0	2.6–5.6			
Cl (meq/L)	Juv	47	103	4	103	95–112	0.79	2, 188	0.457
	Ad	101	102	4	103	94–110			
	Ger	43	102	5	102	92–114			
TCO2 (mEq/l)	Juv	47	16.9	4.9	18.0	5.6–29.4	2.62	2, 188	0.075
	Ad	101	15.9	6.1	17.0	4.0–27.0			
	Ger	43	18.2	5.4	18.0	7.1–31.7			
AGAP (mEq/L)	Juv	47	30.4	6.2	31.0	17.0–44.6	3.48	2, 188	0.033^d^
	Ad	101	33.2	8.1	32.0	19.6–51.9			
	Ger	43	30.2	7.1	29.0	20.1–51.4			
osmo (mOsm/kg)	Juv	47	284.5	6.1	284.0	270.8–301.6	2.84	2, 188	0.061
	Ad	101	287.2	8.2	287.0	272.4–307.2			
	Ger	43	284.5	8.0	285.0	262.5–304.0			
TP (g/dL)	Juv	47	7.3	0.7	7.2	5.6–8.9	2.41	2, 186	0.092
	Ad	99	7.6	0.8	7.6	5.8–9.3			
	Ger	43	7.4	0.9	7.4	5.2–9.7			
ALB (g/dL)	Juv	47	4.5	0.4	4.4	3.5–5.5	8.17	2, 188	< 0.001^b,c^
	Ad	101	4.3	0.6	4.4	2.7–5.4			
	Ger	43	4.0	0.6	4.1	2.8–5.3			
GLOB (mg/dL)	Juv	47	2.8	0.5	2.7	2.1–4.3	15.62	2, 184	< 0.001^a,b^
	Ad	98	3.2	0.5	3.2	2.4–4.5			
	Ger	42	3.3	0.5	3.3	2.3–4.7			
AGR (g/dL)	Juv	47	1.6	0.2	1.6	0.9–2.0	29.55	2, 186	< 0.001^a,b,c^
	Ad	99	1.4	0.3	1.4	0.7–1.8			
	Ger	43	1.2	0.3	1.2	0.4–1.8			
CHOL (mg/dL)	Juv	47	99	24	101	49–148	7.06	2, 187	0.001^b^
	Ad	101	110	30	108	58–166			
	Ger	42	123	35	122	59–198			
TRIG (mg/dL)	Juv	46	135	50	139	58–274	3.91	2, 181	0.022^b^
	Ad	99	158	59	153	67–332			
	Ger	39	170	67	155	64–336*			
FE (μg/dL)	Juv	47	114	32	110	58–199	4.45	2, 186	0.013^b^
	Ad	100	128	41	122	60–228			
	Ger	42	140	49	137	50–290			
TIBC (μg/dL)	Juv	47	397	62	381	302–583	1.32	2, 187	0.269
	Ad	100	379	66	372	247–544			
	Ger	43	390	68	405	233–556			
UIBC (μg/dL)	Juv	47	283	56	272	184–415	5.20	2, 187	0.006^a,b^
	Ad	100	251	65	251	123–414			
	Ger	43	246	66	254	111–356			

*Note:* Value is significantly different between groups (*p* < 0.05; a; juvenile and adult, b; juvenile and geriatric, c; adult and geriatric; d; overall significant difference in values although no significant differences were identified between any two groups).

* Minimum and Maximum from dataset are used in lieu of calculated RI minimum and maximum.

## 4 Discussion

The aim of this study was to produce contemporary hematology and serum chemistry reference intervals using a captive breeding colony of *A*. *nancymae* housed at The University of Texas MD Anderson KCCMR. The results include intervals for each sex and life stage based on robust sample sizes. As KCCMR has the largest breeding colony of *Aotus* spp. in the United States and is a major supplier of these animals for biomedical research, this population is well suited for the generation of updated and expanded reference intervals. While some effects of sex have been previously reported for this species, this is the first published study, to our knowledge, analyzing the effects of aging on hematology parameters and serum chemistry values in a large breeding colony of *A*. *nancymae* [7, 8]. Furthermore, this study generated reference values for a wider range of hematology parameters and serum chemistry analytes than have been previously available for owl monkeys in general. Of note here is that most animal handling at the KCCMR owl monkey breeding colony is performed on conscious, manually restrained animals, without the use of sedatives or anesthetics. This includes every‐other‐month health exams and most study manipulations and blood collections, including the blood samples utilized for this study. Catching and manually restraining animals may cause elevations in some serum analyte values such as CK, LDH, and AST, consistent with myocyte damage. Readers wanting to utilize the reference intervals provided in this report for blood collected from sedated or anesthetized animals should be aware that sedatives and anesthetics may cause notable alterations to some hematological parameters and serum blood analyte values, as has been previously documented in other NHP species [19, 20, 21].

The erythrocyte and platelet reference interval values obtained from our study were found to be highly comparable to those provided in the only other, relatively contemporary, paper on hematological parameters in *A*. *nancymae* [8]. However, the values for WBC, along with the absolute counts for most leukocyte populations, of both sexes were appreciably lower in the KCCMR animals as compared to those from the previous paper. While the exact reason for the leukogram differences between these two sources is unknown, it is notable that while this study utilized blood from captive born, manually restrained animals, the previous paper collected blood from 124 female and 130 male wild‐caught animals that had been sedated with ketamine for blood collections. In short, it is plausible that the increased leukocyte counts seen in the wild‐caught sedated animals could have been due to either increased immune stimulation in the wild‐caught animals as compared to colony‐raised animals, the effects of ketamine sedation on the leukogram, or a combination of both factors [19, 20, 21].

Prior to this report, there were three other, relatively contemporary, papers examining serum chemistry analytes in *A*. *nancymae*. One independently and collectively evaluated 124 female and 130 male wild‐caught animals, one collectively evaluated a mix of 62 male and female animals imported a year prior to the study, and one collectively evaluated a mix of 20 captive‐bred male and female animals for analysis [7, 22, 23]. All three studies utilized ketamine sedation for blood collection. While most of the 14 serum chemistry analyte values that were evaluated through the three previous studies were comparable to those identified in this study, the reference values for TBIL, CHOL, GLUC, PHOS, BUN, and CREA were found to be appreciably higher in at least two, if not all three of those reports. As ketamine has been documented to cause elevating effects in many of these same analytes in other NHP species, and the use of ketamine is the primary difference between the previous studies and this study, ketamine is considered the most likely cause of the differences identified herein [20, 21].

In our current study, we identified statistically significant sex‐related differences in *A*. *nancymae* when the dataset was considered as a whole, without regard to any one age demographic. Specifically, we found that the mean values of HCT, HGB, RBC, CREA, and K were significantly higher in males than females, while PLT mean values were significantly higher in females than males. Previous studies using wild‐caught *A*. *nancymae* have identified statistically significant sex‐related differences in HGB, RBC, and PLT, similar to ours [8]. Additional studies using another owl monkey species (*Aotus azarai infulatus*), capuchin monkeys, marmosets, and macaques have likewise found RBC, HCT, HGB, and/or CREA to be statistically higher in males than females [8, 10, 11, 13, 24, 25, 26, 27]. Collectively, these studies have proposed that the increased mean values of HCT, HGB, RBC and CREAT in male animals is likely primarily due to the increased body size of male, as compared to female, animals, although the differences in hormone production between the sexes may also be at play. The sex‐specific difference identified with the mean values of K in *A*. *nancymae* could potentially represent real differences between the sexes, as similar, mild, sex‐related changes in K have been identified in some rodent studies [28]. However, given the lack of comparable findings in other NHP species, it is equally plausible that this ‘significant’ finding may simply represent an anomaly of the dataset.

When considering the adult owl monkey demographic alone, the mean values of HCT, CREA, and K were significantly higher in males than females, while TRIG mean values were significantly higher in females than males. Although TRIG has not been evaluated as part of the analyte panel in previous owl monkey biochemical reports, there have been similar TRIG findings reported from a few macaque studies [13, 27]. The cause of the significantly elevated TRIG levels in females, as compared to males, has not been elucidated other than it has been proposed that differences in lipid metabolism might be beneficial toward some aspect of reproduction. This proposition is consistent with the finding that this change is found in the adult demographic at KCCMR, the most reproductively active of the demographics, but is not evident in the data set as a whole. The sex‐related differences identified in the adult demographic, as compared to sex‐related differences described above for the entire data set, highlight the importance of examining age as a factor, where possible, when developing reference intervals. Unfortunately, the number of samples available for the juvenile and geriatric demographics in this study were not large enough to provide for a robust comparison between the sexes.

In our comparison of the mean values of hematological parameters and serum chemistry analytes between the juvenile, adult, and geriatric demographics in *A*. *nancymae*, we identified several statistically significant differences between the groups. On the hematological front, HCT, HGB, and RBC were significantly greater in juvenile and adult animals compared to geriatric animals. Moreover, all three parameters showed a general trend toward decreased values with age. The only previous owl monkey study examining age‐related changes to the hemogram did not include geriatric animals and was therefore insufficient for comparison purposes [10]. In comparisons to other species, our findings were found to be opposite those reported for aged macaques, but were consistent with some studies on aging humans, elderly dogs, and our RBC findings were consistent with at least one marmoset study which included aged animals [12, 14, 29, 30, 31, 32]. In the human and dog studies, the most likely causes of these changes were considered to be anemia of chronic disease, gastrointestinal bleeding, or a decrease in erythropoiesis secondary to chronic kidney disease. Chronic nephropathy is a well known cause of morbidity and mortality in owl monkeys in general and has also been associated with hypertension in some studies [33, 34, 35, 36, 37]. Within the KCCMR colony, chronic nephropathy is very common in the geriatric population, and nearly all animals within this demographic are found to have some degree of this condition at necropsy. Milder variations of chronic nephropathy are also sometimes identified in ‘clinically healthy’ adult animals used in studies although the condition is considered very rare in the juveniles within the KCCMR colony. Given this commonality of this condition across the demographics, it is considered plausible that chronic nephropathy in owl monkeys may play a part in the age‐associated erythrocyte changes noted here.

The HCT, HGB, and RBC findings suggest a continued decrease in erythropoiesis with advancing age and this change is suspected to be directly associated with the age‐related differences found for the mean values of FE and UIBC in *A*. *nancymae*. Specifically, as a general trend the mean values of FE show an increase, while the mean values of UIBC show a decrease, with each increasing age demographic. Juvenile mean FE values were significantly lower than those of geriatric mean FE values, and juvenile mean UIBC values were significantly higher than the mean values of adult and geriatric UIBC. No comparable, age‐related NHP studies could be identified that included FE and UIBC as part of their analyses. However, the human literature has identified an inverse correlation between serum FE levels and HCT, HGB, and RBC as greater levels of FE accumulate in the serum when it is not actively being used for erythropoiesis [38, 39]. Given this information, it is considered likely that the increase in mean FE values with advancing age in *A*. *nancymae* is a result of the decreased erythrocyte production over the same time period. The converse decrease in UIBC with age is an expected finding, as with increases in serum FE there will be more FE binding to transferrin and an overall decrease in “unsaturated” binding sites.

Other statically significant, age‐related hematological changes were identified for the EOS%, RDW, MPV, BASO, and BASO% parameters. The mean values of the EOS% showed an inverse correlation with age, and juveniles had significantly greater values than both adults and geriatrics. For RDW, MPV, BASO, and BASO%, the mean values of adults were significantly greater than those of juveniles. Adult mean values for these latter four parameters were also slightly, but not statistically, greater than those of geriatric animals. Comparison of these five parameters to other NHP data is difficult, as they are frequently not included in many age‐related studies. Where NHP data on these five parameters do exist, there appears to be no consistent trends to the age‐related changes between the NHP studies, even those performed using the same species. While the EOS%, RDW, MPV, BASO, and BASO% data represent identifiable changes in the maturation of the hematopoietic system over time, it is notable that the numerical differences between the groups tended to be very small, even between those parameters found to be statically significant. As such, it is plausible that the differences identified here are of minimal clinical importance overall.

There were four unique age‐related findings associated with various serum chemistry analytes in *A*. *nancymae* that have been previously recognized in a variety of other species, including other NHPs. The first age‐related finding here involved the serum protein components. In our study we identified continued decreases in ALB, AGR, and CA associated with advancing age. Geriatric ALB mean values were significantly lower than those of the adult or juvenile values and the mean values of AGR and CA were significantly different between all three demographics. In contrast, we identified a positive correlation between mean GLOB values and advancing age, with the geriatric mean values being significantly higher than those of adults. While no previous age‐related owl monkey studies have included these analytes in their assessments, similar significant differences and trends in ALB, AGR, GLOB, and/or CA have been reported in macaques and marmosets [11, 12, 14, 26, 27, 29, 30]. The decline of ALB with age in other NHP populations has previously been proposed to be due to chronic disease conditions that result in either decreased ALB production by the liver or a loss of ALB through kidney or gastrointestinal sources. Increases in GLOB associated with age have previously been proposed to likely occur as a result of increased antigen exposure and adaptive immunity throughout life, but it is also proposed that similar increased GLOB values could also be expected with ongoing disease processes [27, 32]. Given that decreases in ALB and increases in GLOB are both identified to be associated with advancing age in this population, a plausible cause could be the increased incidence of chronic nephropathy with advancing age in the KCCMR owl monkey colonies, as previously described. The ALB and GLOB values are used to calculate AGR and thereby explain the significant changes to AGR with advancing age. While CA is not a protein component of serum, around 40% of CA is bound to ALB in most species and therefore decreased levels of ALB are known to indirectly lead to decreases in total serum CA [11]. Calculations performed using the mean CA and ALB values from the current dataset to produce mean ‘corrected CA’ values for each age demographic resulted in corrected CA values that were highly comparable between the cohorts. This suggests that there are likely no true age‐related differences in CA here, but since the corrected CA values were calculated using formulas that make several general assumptions about CA physiology across species, the possibility of some age‐related CA differences cannot be definitively ruled out. Direct measurement of ionized‐CA would provide a more accurate assessment of any true CA changes across age demographics; however, this specific assay is not routinely included in the serum chemistry analysis at the KCCMR.

The second well‐recognized, age‐related finding from this study was analyte alterations as a result of musculoskeletal maturation. Specifically, CK, ALP, and PHOS analytes all tended to have a decrease in mean values with age. Mean juvenile values of CK were identified to be significantly greater than adult and geriatric mean values, while the mean values of both ALP and PHOS were significantly different between all three age demographics. Similar trends with regard to an age‐related decline in CK are identified in the human and veterinary literature and include at least one contemporary macaque paper [13, 40, 41]. Most serum CK in healthy animals is appreciated to originate from muscles during growth, damage, and repair. As muscular growth, differentiation, and turnover are all highest in young animals and decline with age, the mean values of CK can likewise be expected to be highest in the juvenile demographic with declining values expected with advancing age. With regard to our ALP and PHOS findings, similar significant differences and trends have also been reported in humans and a wide variety of animals, to include other owl monkey species, macaques, and marmosets [10, 11, 13, 14, 26, 27]. ALP and PHOS are generally recognized to be elevated in juveniles as a result of the high osteoblastic activity of bone growth in young animals, and both analytes tend to decrease with age as regular bone growth and turnover become less prominent. As CA is also involved in bone growth and can sometimes follow similar age‐related patterns as is found with ALP and PHOS, it is possible that the age‐related decline in CA identified here may also be in part due to changes in bone turnover. However, since the decreases in CA correlate with the decreases in ALB, as discussed above, it is considered unlikely that the age‐associated CA changes here are completely due to changes in bone turnover alone.

The significantly increased mean values in CREA identified in adult and geriatric animals, as compared to juvenile animals, constitute the third finding in this study that has been previously well‐described in other species. Specifically, similar significant differences and trends in CREA have previously been reported in another owl monkey species, macaques, and marmosets [10, 12, 26, 27]. The primary cause of this change in most age‐related reference interval studies is proposed to be the increased muscle mass of matured animal populations as compared to the juvenile animals, although some studies have also suggested decreased renal function with age as a potential cause as well. While it is possible that chronic kidney disease could play some part in this change here, increased muscle mass of the matured animals is considered the primary reason for the demographic differences in the KCCMR dataset. This conclusion is largely based on the finding that, even though age‐related chronic kidney disease does occur frequently in this population, the BUN data do not suggest any notable deficiencies in the renal filtration with increasing age and, furthermore, there were no significant differences in the CREA mean values between the adult and geriatric population. With regard to the BUN changes in this dataset, the mean BUN value of juveniles was identified to be significantly higher than the mean BUN value of adults, which is numerically similar to geriatric animals. Similar significant differences and trends showing a decrease in the mean BUN value between juvenile and adult animals have previously been reported in some macaque studies [13, 14]. Lacking other explanations, the BUN changes presumably represent differences associated with the maturation of the juvenile renal filtration process.

A generalized increase in CHOL and TRIG with increasing age was identified in the KCCMR dataset, with both analytes having significantly higher mean values in geriatric, as compared to the juvenile, animals. These changes represent the fourth well‐recognized age‐related finding in this study, as similar significant differences and trends in CHOL and/or TRIG have been previously reported in macaques and marmosets [11, 13, 14, 27, 29, 30]. While no formal mechanism to explain these changes has been proposed, it is plausible that the trend toward an increase of either CHOL, TRIG, or both analytes with age represents a natural shift in metabolic function over time, with geriatric animals eventually leaning more toward a negative energy balance.

Finally, we identified age‐related serum chemistry analyte changes for GLUC and GGT in *A*. *nancymae* that appear to be unique to this study. For GLUC, juvenile mean values were significantly lower than those of adult mean values, which were numerically similar to geriatric animals. Significant age‐related glucose differences were not commonly identified in previous macaque and marmoset studies; however, there are at least two NHP studies that demonstrate a trend for GLUC values to be highest in juvenile animals with a decrease in values associated with increasing age, in direct contrast to our findings [11, 12, 13, 14, 27]. While our GLUC finding could just represent an anomaly of the data set, it is also considered possible that this finding is related to the blood collection process utilized at KCCMR. Animals within the KCCMR colony are usually manually captured and restrained for blood collections without the use of sedatives or anesthetics. This is generally considered advantageous to the overall health of the animals as most mature animals become very habituated to the handling process through the basic health exams and treatments that occur every other month. However, prior to being habituated, young animals generally resist capture and struggle more when restrained than mature animals do. Given that this excessive activity requires increased glucose usage, and increased glucose utilization is recognized as a primary cause of decreased serum glucose in healthy young people and animals, we believe this to be a plausible explanation for the significantly decreased GLUC identified in juvenile animals as compared to other demographics [42, 43, 44] It is also notable that while our results differ from all previous age‐related NHP studies that have included GLUC, all those previous studies have utilized blood collected from ketamine‐sedated animals. Future studies may compare the effects of conscious manual restraint to alternative anesthetic regimens to assess the clinicopathologic differences and potential welfare indications of each collection method.

The GGT findings from this study identified a significant difference between all three age demographics of *A*. *nancymae* with an overall increase in mean GGT values with each advancing age group. This finding is in direct contrast to other age‐related studies in macaques which identified trends toward an overall, sometimes significant, decrease in GGT with age [13, 14, 27, 45]. The underlying cause of this discrepancy is unknown but cholestatic disease in the aged *A*. *nancymae* is not considered likely as other analytes that might be associated with an increased incidence of age‐related biliary and/or liver disease (TBIL, ALP, ALT, AST, etc.) do not reflect similar changes. Given that tissue expression of GGT is known to vary by species, and in some species can include renal epithelium, it is tempting to propose that this age‐associated increase in GGT could be in some way connected to the incidence of chronic renal disease in this colony [46, 47]. However, without investigating the prominence of GGT in owl monkey renal epithelium and, if present, proving its effect on serum GGT, this last suggestion remains pure speculation.

In conclusion, this study utilized blood collected from conscious, manually restrained, male and female *A*. *nancymae* of various ages to produce the most complete set of hematology and serum chemistry reference intervals for this species to date. The mean values of these data were compared between the sexes and across three clinically relevant age demographics, and we have identified a number of statistically significant differences within each comparison. For the vast majority of the sex‐related and age‐related differences identified in this report, we have identified similar findings within the literature from other NHP species. In the few instances where our sex‐related and age‐related findings were not supported by previous NHP studies, we have tried to provide plausible explanations as to a possible cause of these novel findings. We believe this resource will be of great utility to the research community as a whole when used as a reference tool for captive bred, research naïve, manually restrained *A*. *nancymae*. However, caution in interpretation of these reference intervals should be warranted when extrapolating these data to owl monkey species other than *A*. *nancymae* and/or when animals do not meet the aforementioned criteria of being captive bred, research naïve, and manually restrained. Furthermore, there is a plethora of environmental and sample collection factors that may influence hematological results, and as such, best practice may be the generation of colony‐specific blood/serum reference ranges.

## Funding

The authors have nothing to report.

## Ethics Statement

The authors confirm that this study adhered to the ethical policies of the journal, as noted on the journal's author guidelines page. Animal studies were performed at the University of Texas MD Anderson Cancer Center's Michale E Keeling Center for Comparative Medicine and Research and complied with international, national, and institutional guidelines for humane animal treatment, including those set forth by US National Research Council “Guide for the Care and Use of Laboratory Animals” and the US Public Health Service “Policy on Humane Care and Use of Laboratory Animals” and the Animal Welfare Act. All studies were conducted following approval of the MDACC Institutional Animal Care and Use Committee (protocol # 0329‐RNXX).

## Conflicts of Interest

The authors declare no conflicts of interest.

## Data Availability

The data that support the findings of this study are openly available in Open Science Framework at https://doi.org/10.17605/OSF.IO/GVJDY.
